# Postoperative thromboembolic prophylaxis in joint replacement surgery: Guidelines and daily practice

**DOI:** 10.1186/1477-9560-10-18

**Published:** 2012-09-03

**Authors:** Arina J ten Cate-Hoek, Karly Hamulyák

**Affiliations:** 1 Department of Internal medicine, Maastricht University Medical Center and Cardiovascular Research Institute (CARIM), Maastricht, the Netherlands

## Abstract

This is a commentary discussing the article published in Thrombosis Journal by Subramanian et al. [Thrombosis Journal 2012, 10:15].

## 

The need for post-operative thromboembolic prophylaxis in orthopaedic surgery is beyond debate. Post-operative thrombosis is prevalent in these patients. In the absence of thromboprophylaxis, the incidence of venographically confirmed deep vein thrombosis (DVT) in the first 7 to 14 days following surgery is about 40-60%. About half of these thrombi become symptomatic. In most cases DVT is diagnosed within a period of two months after the patients’ discharge from hospital [1].

Guidelines on prophylaxis of venous thromboembolism that have been published by at least four different organisations established in the field of medical research, all agree on the need for post-operative prophylaxis [2-5].

Current prophylactic treatment is highly effective, and a significant reduction in the venous thromboembolism (VTE) incidence can therefore be reached by the use of adequate prophylaxis [6,7].

However, despite the various guideline recommendations and readily available efficacious medication, the use of preventive measures against VTE in clinical practice has been found to be sub-optimal in several instances. A retrospective study by Yu *et al. *[8] found that the overall compliance with (ACCP) guidelines for the prevention of VTE in six American hospitals was only 13.3%, ranging from 2.8% for neurosurgery to 52.4% for orthopaedic surgery. A survey amongst surgeons in the United Kingdom by Sharif *et al. *[9] showed that only 7% of respondents were adhering to NICE guidance after hip fracture surgery and 44% after hip arthroplasty.

Therefore, the stated rationale and considerations by the authors of the article published *Thrombosis Journal* on the 12 month review of a modified protocol using low dose Dabigatran etexilate in postoperative thromboembolic prophylaxis in joint replacement surgery, to deviate from the NICE guideline, seem legitimate [10]. On the one hand, the deviation from the guidelines is in favour of increased compliance with protocol by in-hospital (junior) staff. On the other hand, enhanced patient compliance and adherence in the out of hospital setting is anticipated by avoiding the need for self administered injections at home.

Usually guidelines fall behind with innovations in clinical practice. After the introduction of new treatment modalities some time passes before synthesized evidence finds its way into guideline recommendations. In the case of the novel oral anticoagulant drugs such as dabigatran and rivaroxaban this implementation into guidelines has been relatively swift.

At this moment there is lack of day-to-day clinical experience with the newly introduced drugs and therefore clinical evidence is less unequivocal. Clinical practice usually differs from the setting of a clinical trial. Post surgical gastrointestinal upset and vomiting may reduce the effectiveness of oral antithrombotic therapy. To start prophylactic treatment post surgery with subcutaneous administered LMWH is therefore an attractive alternative.

The choice of the authors for a combination treatment of two individually established safe and efficient therapeutic treatment modalities is understandable, but has a number of methodological shortcomings. Patient management was instituted without any preliminary studies, such as to identify the optimal, safe and effective, dosing strategy for the overlap of two different treatment modalities. In addition, a pragmatic, non-evidence based, choice was made for the single use of a low dose of 150 mg dabigatran once daily, irrespective of patient characteristics such as bodyweight, age or kidney function. On the other hand, all consecutive patients who met the inclusion criteria were managed according to the protocol, minimising selection bias. Data on complications such as VTE, bleeding, transfusion rates, periprocedural infections and mortality were prospectively collected and reviewed by an independent reviewer to control for bias.

In parallel to the article by Subramanian *et al.*, comparable clinical experience was gained in the United States, in this case in the setting of a clinical study, with similar alternative treatment strategies, bridging the periprocedural period with LMWH and making the transition to oral anticoagulant treatment before hospital discharge [11]. This study investigated the optimal interval for a safe overlap in anticoagulant activity between the LMWH and the oral anticoagulant drug rivaroxaban. The underlying rationale for the study being essentially the same: adapting the strategy to the requirements of day-to-day clinical practice.

The need for (observational) data on the novel oral anticoagulant drugs derived from clinical practice is widely recognized. Despite methodological shortcomings, the recent Subramanian *et al.* article adds to our knowledge on the everyday use of the novel oral anticoagulant medication and shows that the choice of prophylactic treatment does depend on clinical circumstances but also on patient preference.
